# Spectral Analysis of Macro-Fiber Composites Measured Vibration of Double-Panel Structure Coupled with Solenoids

**DOI:** 10.3390/s20123505

**Published:** 2020-06-21

**Authors:** Anna Chraponska, Jaroslaw Rzepecki, Chukwuemeke William Isaac, Krzysztof Mazur, Marek Pawelczyk

**Affiliations:** Department of Measurements and Control Systems, Silesian University of Technology, Akademicka 16, 44-100 Gliwice, Poland; jaroslaw.rzepecki@polsl.pl (J.R.); chukwuemeke.william.isaac@polsl.pl (C.W.I.); krzysztof.jan.mazur@polsl.pl (K.M.); marek.pawelczyk@polsl.pl (M.P.)

**Keywords:** double-panel structure, solenoid coupling, rigid device casing, vibration control, macro-fiber composites, spectral analysis

## Abstract

Noise may have a negative impact on humans health and well being. Noise is a direct result of the vibration of structures. Many industrial workers and people using household appliances may be exposed to these harmful factors. To minimize their negative consequences, different approaches to noise and vibration reduction may be applied, e.g., active, semi-active or passive methods. In this research, a semi-active approach to vibration reduction of a cubic rigid casing enclosing a noise- and vibration-generating device is presented. One of the casing walls consists of double thin steel panels, coupled with the use of electromagnetic dampers—solenoids installed in the space between the panels. Other casing walls are built of single plywood panels. Vibrations of the outer (radiating) panel of the wall are measured by Macro-Fiber Composite patches. Spectral analysis of structure vibration is carried out to identify the benefits of the proposed coupling solution in terms of vibration reduction of the wall. The frequency range, where vibration reduction is observed, depends on the number of activated solenoids and the duty cycle of a Pulse Width Modulation (PWM) signal. Advantages and drawbacks of the proposed method are discussed and future improvements of the examined setup are suggested.

## 1. Introduction

Noise exposure may have a negative impact on humans health and well being, as it may lead not only to hearing damage, but also to non-auditory adverse health effects [1]. The risk of health damage depends on noise frequency and the duration of noise exposure [2]. This issue is mainly observed in the industrial work environment, where uncontrolled exposure to noise produced by machines may result in hearing disfunctions of employees [3]. Environmental noise is also examined in the context of transportation, as high levels of aircraft, road, wind turbine [4] and railway noise may lead to health disturbances [5]. For instance, railway traffic noise levels can be dangerous for humans in an urban environment [6]. As the recent studies show, railway noise coming from different sources cannot be neglected in modeling due to its impact on humans [7]. Aircraft overflights noise may lead to biodiversity loss [8]. Recently, coastal and port areas have also been investigated as port activities have been identified as an important source of noise [9]. Different possible effects of environmental noise have been well recognized, based on existing studies [10]. Noise is a direct consequence of vibration of structures.

Human exposure to vibration, mainly observed in transport and industry, is especially dangerous at low frequencies, up to 100 Hz [11]. Different possible sources of vibration-induced health damage exist. Handheld vibrating machines may lead to skeletal or muscular pathologies [12]. Floor vibration may also have harmful effects on office workers in buildings [13].

The common goal of researchers in the field of noise and vibration control is to effectively reduce exposure to noise and vibration in the environment. Different materials and methods may be employed, depending on the examined case. For instance, if road traffic is considered, Bus Signal Priority (BSP) may be employed as an urban noise reduction solution [14]. Road traffic noise prediction models help the researchers investigating health outcomes worldwide [15]. Modeling of low-noise pavements is also crucial due to the influence of their acoustic performance on urban noise levels [16].

In the field of noise reduction, three main methods are widely recognized: passive, semi-active and active.

Passive approaches to noise control allow for noise attenuation by using different types of enclosures, barriers and silencers, while in passive vibration control, spring-mass-damper decoupling can be employed [17]. Damping is also important, for instance, in the systems measuring pavements’ acoustic impedance, to ensure its correct evaluation [18]. Both passive noise and vibration control techniques are effective only at the mid- and high frequencies [19]. At low frequencies, passive noise barriers’ dimensions, mass, and cost should be increased, which makes them unaccepted for many applications [20].

Active methods, despite their high cost and complexity [21], are a lightweight solution to low-frequency sound and vibration problems [22]. In a classic approach, they introduce controllable secondary sources, which are driven to produce the output interfering destructively with primary source disturbance [22]. Another approach may be Active Structural Acoustic Control, which employs shakers or piezoelectric patch actuators [23] as secondary sources.

Hybrid techniques combine benefits of passive and active techniques [21]. One of the examples is a nonlinear electronic damping technique, Synchronized Switch Damping (SSD), which is robust to environmental variation [24].

One of the approaches to noise and vibration reduction may be a casing, which encloses a noise-generating device. Such casing may differ in its design and properties. In previous research, the authors proposed, inter alia, one of SSD’s variants [25] as a robust semi-active approach in case of a rigid device casing, and an Active Structural Acoustic Control approach to control a lightweight active casing placed at a wall [26] or in a corner [27].

In this research, a semi-active method is employed for reduction of the double-panel structure vibration. Such a method is called semi-active, as the actuator is supplied with the energy source to change properties of the structure, and not to force a vibration [28]. A noise-generating loudspeaker is placed inside the cubic rigid casing. Each of the casing walls may be built of single- or double panels [29]. In this study, one of the casing walls is a double-panel structure, consisting of thin steel panels, coupled with the use of electromagnetic actuators—solenoids. Each solenoid’s stiffness may vary, depending on the duty cycle specified with the use of a Pulse Width Modulation method. The other walls of the casing are built of plywood single panels. The details of the rigid device casing are presented in Section 2.1.

The double-panel structures are widely used due to their property of a good sound insulation [20]. As they are also characterized by a light weight, they are widely used in aerospace [30], railway, automotive, and other industries [31]. However, their performance decays at low frequencies mainly due to the mass-air-mass resonance, where, in turn, an active control approach may be applied [32,33]. One of the advantages of a double-panel structure over a single panel is that its sound-absorbing performance can be improved, e.g., by interlayers and absorbing materials [34,35]. To improve structure performance around the mass-air-mass resonance, a mass-spring-damper system may be applied [36]. In recent years, research on the orthogonally stiffened double composite panel structures has also been done [37,38].

In this study, Macro-Fiber Composite (MFC) patches are attached to the radiating panel of the double-panel structure to measure vibration of this panel. Piezocomposites, possible to use both as sensors and actuators, are known for their high-quality properties, such as low mass and high flexibility [39]. This is an important class of smart materials, which produce electrical charge if exposed to mechanical deformations [40]. In this research, five MFC elements are used to measure vibration of the radiating panel of the double-panel structure. Positions of these elements were arbitrary selected. Experimental results are analyzed to determine, what is the influence of such location of the piezocomposites on the overall performance of the structure. However, to get more benefits from the method, placement of MFC elements should be a subject of optimization, to guarantee that all the considered vibration modes are observed and the noise-to-signal ratio is reduced.

As in Lalanne [41], random vibration may be analyzed either by applying statistical methods to the signals with respect to time or by plotting signal spectra. There exist several parameters useful in such analysis, e.g., Root Mean Square value of the signal or Power Spectral Density (PSD). In this research, vibrations measured by Macro-Fiber Composite sensors are presented in the frequency domain as the Power Spectral Density estimates of the input signals and respective conclusions are drawn.

The remainder of this paper is organised as follows: in Section 2 the experimental setup is presented and the rigid device casing is desribed in details. The choice of placements of MFC sensors and electromagnetic dampers (solenoids) is explained. A description of the research experiment is included. In Section 3, results are presented in the frequency domain, and in Section 4 they are discussed in details. Section 5 contains observations and conclusions from the research. It presents advantages and drawbacks of the proposed method, and discusses possibilities for the future development of the presented method.

## 2. Materials and Methods

### 2.1. Experimental Setup

**Figure 1:**
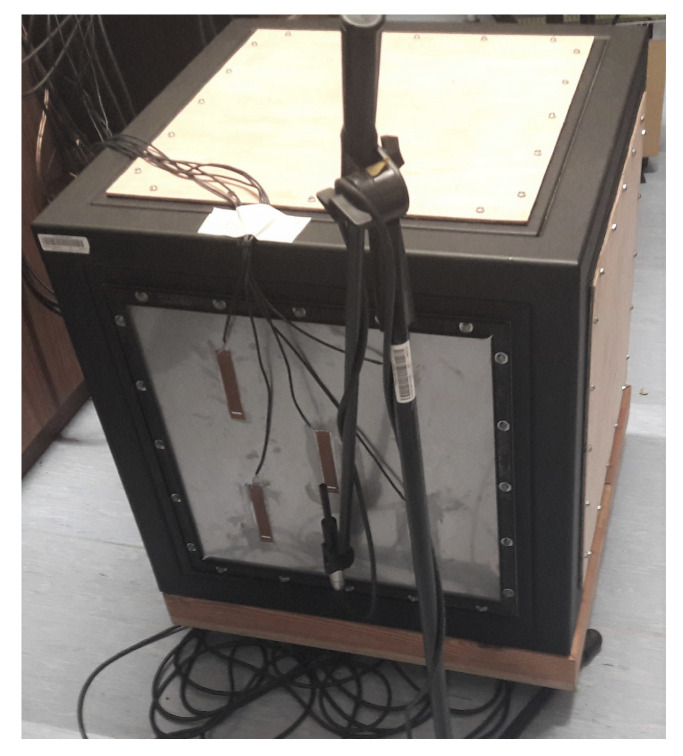
The experimental setup [42].

The experimental setup is shown in Figure 1. It consists of a rigid device casing, a noise-emitting loudspeaker placed inside the casing, the solenoids between panels of casing’s front wall being a double-panel structure, and MFC elements measuring vibration of its radiating panel. Descriptions of particular objects are provided below.

The rigid casing employed in this research has a cubic shape. Its dimensions are 600 mm × 600 mm × 600 mm [29]. Each casing wall, except the sound-insulated base, consists of single or double panels mounted to the heavy frame with twenty screws. Such assembly allows to obtain approximately boundary conditions known as “fully clamped” [43]. In such case, if a double-panel structure is fully clamped, each panel’s moment rotation and transverse deflection along its edges are assumed to be zero [44]. If each panel has the dimensions of a×b, then transverse displacements $w_{1}$ and $w_{2}$ may be expressed as [44]:

(1) $$x\in \left\{0,a\right\},\;0\leq y\leq b,\;w_{1}=w_{2}=0,\;\frac{\partial w_{1}}{\partial x}=\frac{\partial w_{2}}{\partial x}=0;$$

(2) $$y\in \left\{0,b\right\},\;0\leq x\leq a,\;w_{1}=w_{2}=0,\;\frac{\partial w_{1}}{\partial y}=\frac{\partial w_{2}}{\partial y}=0.$$

Each panel has the dimensions of 420 mm × 420 mm. Left, right, back and top casing walls are built of single panels, while its front wall consists of double panels. The single-panel walls are made of plywood, and additional layer of 40 mm thick foam is attached to their inner sides, to increase their sound-insulating properties.

The double-panel structure consists of thin steel panels. The distance between the panels is equal to 50 mm. The inner (incident [20]) panel is 0.6 mm thick, and the outer (radiating [20]) panel is 0.5 mm thick. The double-panel structure considered in this research is an asymmetric one, because such design provides different resonant frequencies of the panels. In such case, if an active control system is applied, the issue of lack of observability in the air cavity of the structure can be easily avoided [45]. Between the panels, five electromagnetic dampers (solenoids) are mounted perpendicularly to each panel’s surface. The solenoid’s properties are given in Table 1.

Each solenoid consists of a coil and a ferromagnetic core. When the current flows through the coil, an electromotive force is induced inside, which causes the core to be held inside the coil’s centre. The average electromotive force depends on the average current, which is set with the use of PWM. Peak-to-peak voltage is equal to 11 V. The maximal voltage value (12 V), specified for a solenoid in Table 1, was not applied during the experiments due to safety reasons.

**Figure 2:**
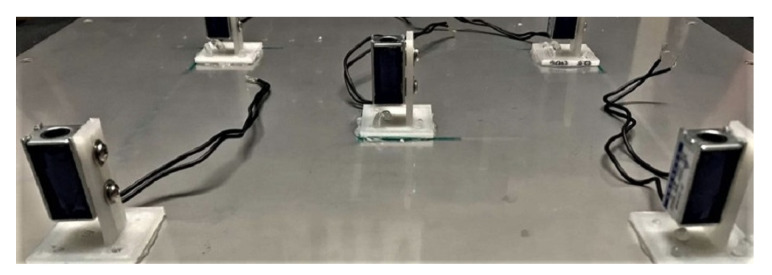
The solenoid coils attached to the incident panel.

The coils are mounted to the outer side of the incident panel with the use of dedicated elements, printed using 3D technology (Figure 2). The cores are mounted to the inner side of the radiating panel. Each core is placed inside the corresponding coil’s centre.

**Figure 3:**
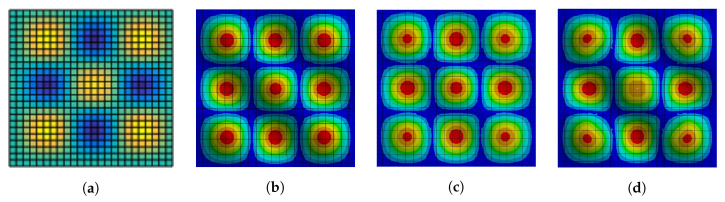
(**a**) Simulated mode (3,3) shape of a single panel. (**b**) Numerical model of the radiating panel’s mode (3,3) shape without couplings. (**c**) Numerical model of the radiating panel’s mode (3,3) shape with 5 inactive couplings. (**d**) Numerical model of the radiating panel’s mode (3,3) shape with five activated couplings.

On the outer side of the radiating panel, five MFC M8514-P2 elements were attached to its surface with the use of epoxy glue. Each MFC element’s performance was checked before the experiment with the use of reference input to ensure stable measurements. However, quality of the MFCs installation may slightly vary along the panel surface. The authors assumed that the differences do not affect the results significantly, as the use of epoxy glue provides rigid connection between the MFCs and the panel. As there are five sensors and five dampers in the experimental setup, their distribution was assumed to be regular in this stage of research. To determine exact placements of MFCs and solenoids, the mode (3,3) shape was used because it is characterized by exactly five highest amplitude peaks in the same phase. In Figure 3, mode (3,3) shapes for a single panel (Figure 3a) and for the radiating panel of the double wall (Figure 3b–d) are compared [42].

Figure 3b–d present three different numerical models of the double-panel structure, implemented with the use of ANSYS software. In Figure 3b, numerical model of the radiating panel’s mode (3,3) shape without couplings is presented. In this case, there is only the air cavity between the panels. In Figure 3c, numerical model of the radiating panel’s mode (3,3) shape with 5 inactive couplings is presented. In this case, the solenoids are considered as the mass loadings only. In Figure 3d, numerical model of the radiating panel’s mode (3,3) shape with five activated couplings is presented. In this case, the solenoid coils and cores are considered as the mass loadings connected with the springs characterized by the assumed stiffness. Although presented mode shapes are slightly irregular for the double wall, they are similar to mode shape of the single panel.

**Figure 4:**
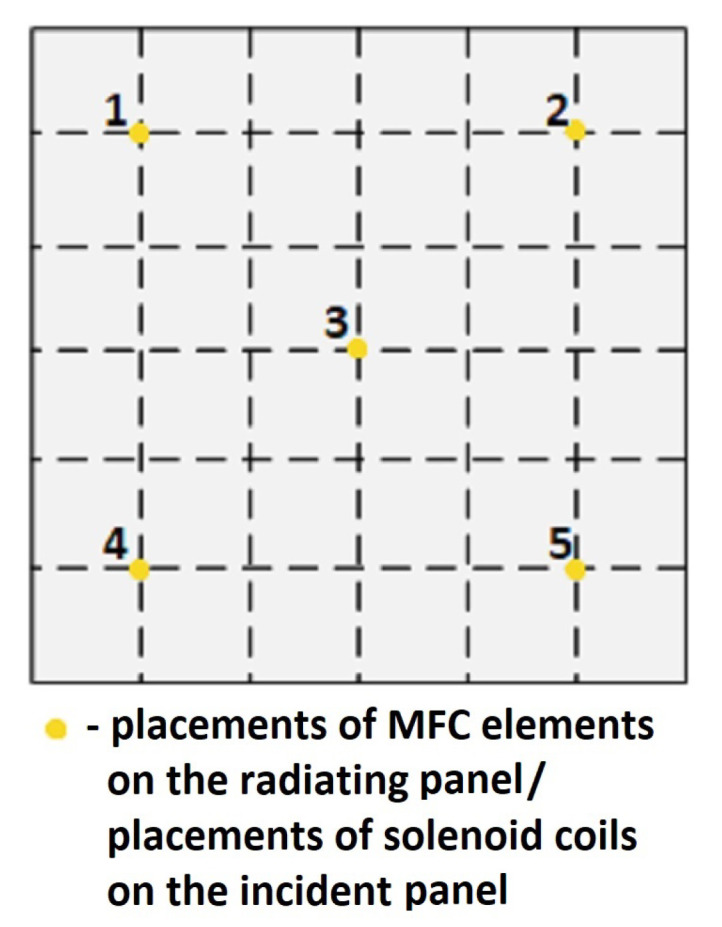
The schematic representation of the MFCs and solenoids placements on the radiating panel and the incident panel, respectively.

Based on the models outcome, placements of MFCs and solenoids on the panels were specified. The schematic representation of both the incident panel and the radiating panel is presented in Figure 4. The exact placements of solenoids and MFCs are marked as the yellow dots. Each solenoid/ MFC is numbered from 1 to 5, as in Figure 4. Hence, in further analysis provided below in this paper, the following nomenclature for MFC elements is used: MFC1, MFC2, MFC3, MFC4 and MFC5. The following nomenclature is used for the solenoids which couple the panels: C1, C2, C3, C4 and C5.

It is noteworthy that such coordinates of sensors and dampers on the panels may not be the optimal ones in terms of controllability and observability measures defined for the considered noise frequency band. The preliminary experiment is performed to identify the benefits of the presented double-panel structure modification, whether the interconnection can support reduction of noise or vibration transmitted through the structure to the environment. It is a starting point for the future optimization of solenoids’ and MFC elements’ placements.

**Figure 5:**
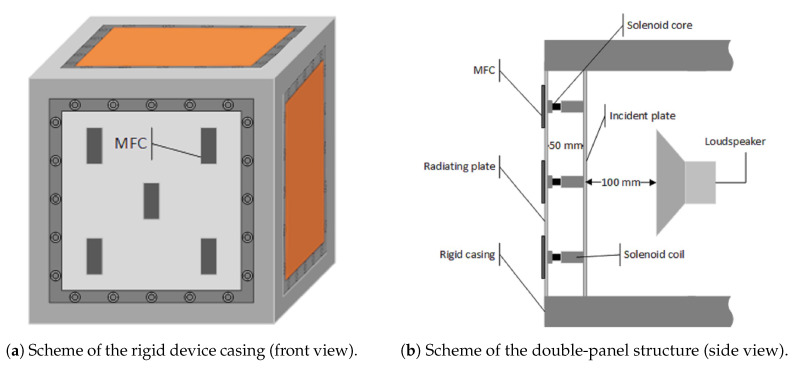
Schematic representation of the experimental setup.

Inside the casing, an active loudspeaker is placed as the noise source. The loudspeaker emits broadband noise up to 500 Hz. Its membrane is placed at a distance equal to 100 mm from the incident panel of the double-panel structure. A simplified schematic representation of the experimental setup is presented in Figure 5.

### 2.2. Research Experiment

The research experiment was performed in several series. MFC elements were used to measure vibration of the radiating panel, when the double-panel structure was excited with a broadband noise (up to 500 Hz) emitted by the loudspeaker. The solenoids acted as the dampers, if voltage was applied. A PWM signal, used to supply the solenoids, was characterized by peak-to-peak voltage equal to 11 V, and its duty cycle was modified with the use of PWM method. If a solenoid was charged, it was considered as activated, as applying current to solenoid’s coil induces an electromotive force which causes ferromagnetic core to be held inside the coil’s centre. If the solenoid was not charged, and only acted as a mass between two panels, then it was considered as inactive.

Four main scenarios were considered for different numbers of activated solenoids. The first scenario was the reference, when all solenoids were inactive and the core was able to move inside the coil, as an electromotive force was not induced. In the second scenario, one solenoid (numbered 3, the central one) was activated. In the third scenario, four solenoids (located near the panel’s corners, numbered as 1, 2, 4, and 5) were activated. In the fourth scenario, all solenoids were activated.

Each of measurement series for these scenarios was repeated for different values of duty cycle of the PWM signal: 25%, 50%, 75%, and 99%. Hence, there were thirteen measurement series in total, including the reference (Table 2). The change of the duty cycle was justified by its indirect influence on stiffness of panels couplings. As applying voltage characterized by 99% duty cycle causes the solenoid’s coil to heat up quickly, lower values of duty cycle were also checked, to observe if it is possible to achieve vibration reduction on the similar level when setting up a lower duty cycle of a PWM signal.

Software used for elements control and raw data acquisition was implemented with the use of LabVIEW environment. A program was developed to generate an excitation signal, to modulate a PWM signal supplying the solenoids, and to acquire measurement data from MFC elements with sampling rate equal to 20 kHz. Raw data from MFC elements, as the vectors of samples, were further processed with the use of MATLAB^®^ environment.

## 3. Results

As stated in Section 2.2, raw data measured by MFCs were collected as vectors of samples of length 560k, which contained 28 series of measurements. First 20k samples were discarded. Data were further processed to obtain vectors of length 20k, averaged from 27 measurement series. The data were transformed into frequency domain to observe the frequencies and amplitudes of the peaks.

Random vibration may be analyzed either by applying statistical methods to the signals with respect to time or by plotting signal spectra [41]. In this research, vibrations measured by Macro-Fiber Composite sensors are presented as Power Spectral Density estimates of the input signals, obtained with the use of the Welch method, where sampling rate is 20 kHz, expected PSD spectral resolution is 1 Hz, Hanning window’s length is 5k, and number of overlapped samples is 0.

**Figure 6:**
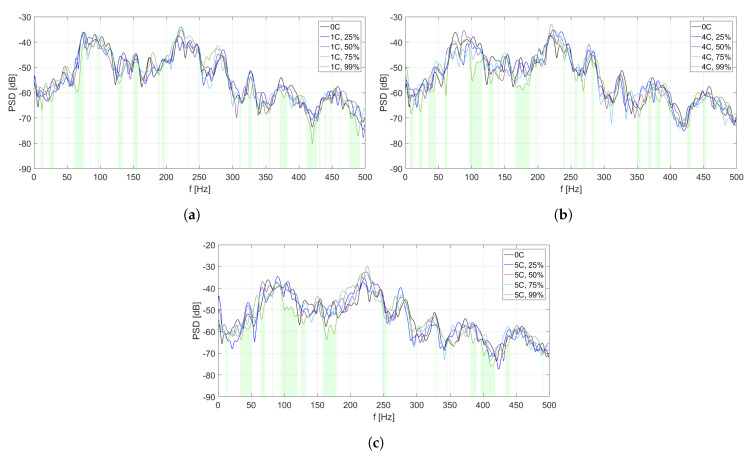
PSD estimates of MFC1 input signals: (**a**) scenario of one coupling activated, (**b**) scenario of four couplings activated, (**c**) scenario of five couplings activated.

**Figure 7:**
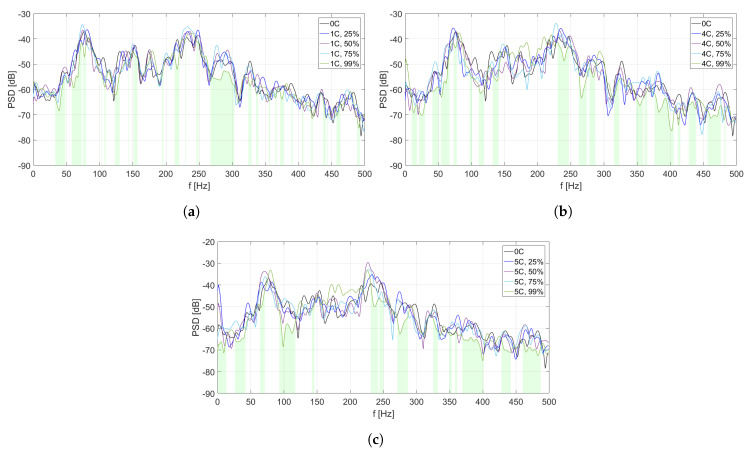
PSD estimates of MFC2 input signals: (**a**) scenario of one coupling activated, (**b**) scenario of four couplings activated, (**c**) scenario of five couplings activated.

**Figure 8:**
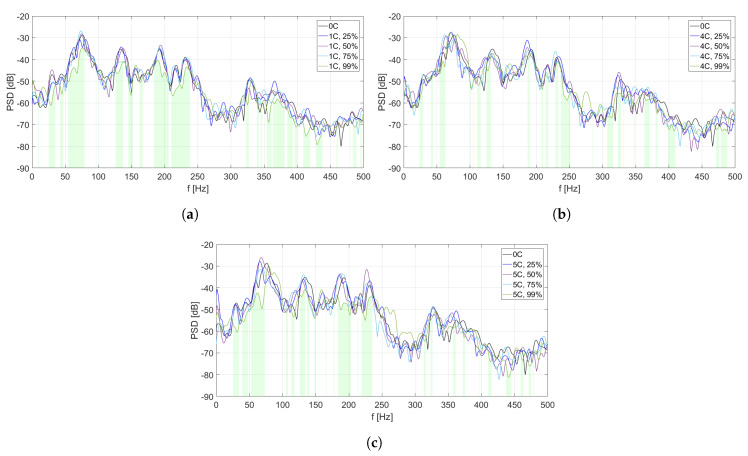
PSD estimates of MFC3 input signals: (**a**) scenario of one coupling activated, (**b**) scenario of four couplings activated, (**c**) scenario of five couplings activated.

**Figure 9:**
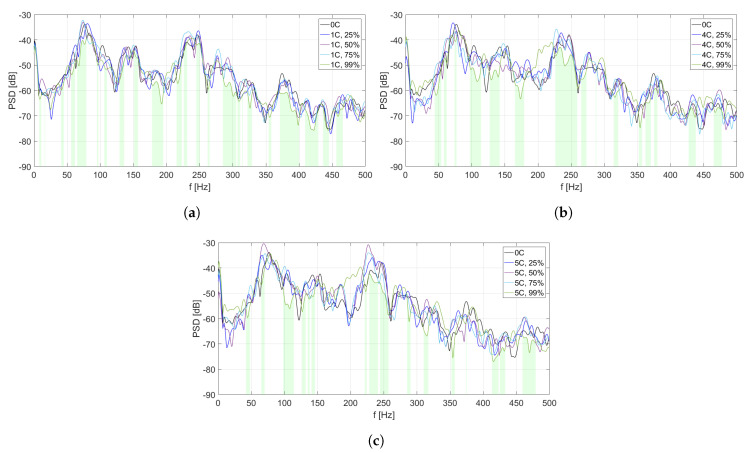
PSD estimates of MFC4 input signals: (**a**) scenario of one coupling activated, (**b**) scenario of four couplings activated, (**c**) scenario of five couplings activated.

**Figure 10:**
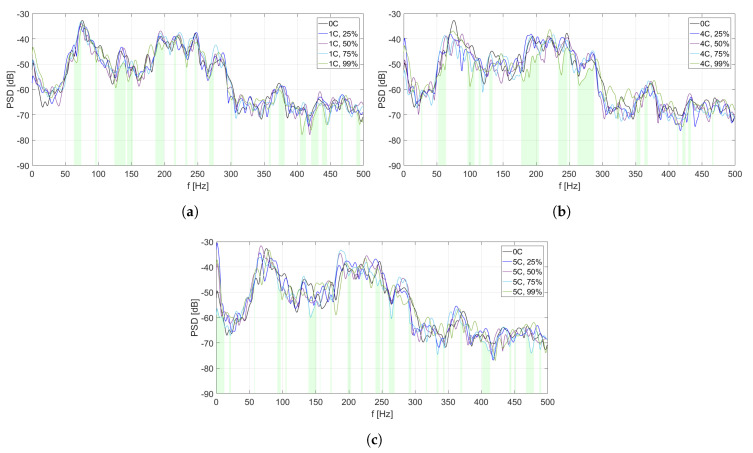
PSD estimates of MFC5 input signals: (**a**) scenario of one coupling activated, (**b**) scenario of four couplings activated, (**c**) scenario of five couplings activated.

The analysis of PSD estimate for each MFC element is divided into two parts. In Figure 6, Figure 7, Figure 8, Figure 9 and Figure 10, PSD estimates are plotted separately for different number of activated solenoids, in the frequency range up to 500 Hz. For each specified number of activated solenoids, the reference scenario, with all solenoids considered as inactive, is also included. Each Figure shows an influence of variable duty cycle on the obtained PSD estimates of the input signals.

In the second part of the analysis, Table 3, Table 4, Table 5, Table 6 and Table 7 are provided to present the most efficient value of duty cycle for each scenario. The efficiency factor for a specific coupling, expressed in %, is calculated as a percent of PSD estimate’s frequencies in range 0–500 Hz, where a specific duty cycle leads to the lowest PSD estimate. In the calculations, PSD spectral resolution equal to 1 Hz is assumed. For instance, if one active solenoid is considered, the efficiency factor is calculated for the reference scenario and for all specified values of duty cycle (from 25% to 99%). As five different scenarios are considered, efficiency factor is calculated for each of them in the given PSD estimate’s frequency range, and sum of efficiency factors should always be equal to 100%, as each frequency value is considered during the calculation process. Validation of obtained efficiency factor values is provided in the corresponding Table. For better readability of the tables, the highest value of efficiency factor in each scenario is in boldface. The reference scenario is included in all calculations as it is assumed that for some frequencies it may be more beneficial not to turn on some of the solenoids in view of the level of possible vibration reduction.

Figure 6, Figure 7, Figure 8, Figure 9 and Figure 10 and Table 3, Table 4, Table 5, Table 6 and Table 7 are related to each other. In each of the mentioned Figures, the areas below curves of the PSD estimates providing the highest efficiency factor values are filled with light green color, to visualize the performance of the best scenarios, based on the calculated efficiency factors.

In this analysis, maximal differences between the lowest and highest PSD estimates in each Figure are not included, as their values may change due to change of Welch method parameters, and cannot be considered as an absolute value of vibration reduction. This research aims to focus on the overall benefits of double-panel structure employment, and to provide a justification for the use of electromagnetic couplings of the panels, as it is a basis for a future development of the proposed method, also with other coupling elements.

## 4. Discussion

### 4.1. Coupling Point No. 1

In Figure 6, different PSD estimates of the input signals from MFC1 are presented in the scenarios of one coupling activated (Figure 6a), four couplings activated (Figure 6b), and five couplings activated (Figure 6c), respectively. In each scenario, four different values of duty cycle of a PWM signal are considered: 25%, 50%, 75%, and 99%. As the reference scenario, PSD estimate of MFC1 input signal in case of all couplings being inactive is included in every figure.

In Figure 6c (five couplings activated), the levels of PSD estimates are in general sligthly higher than in Figure 6a,b. However, in this scenario, the widest frequency bands of vibration reduction are observed, especially around 100 Hz and over 150 Hz, if the duty cycle is set to 99%. A similar effect is observed for a scenario of four couplings activated (Figure 6b). However, for the considered coupling point, many frequency ranges are not marked if the duty cycle value is 99%. Hence, performance around this coupling strongly depends on changes of its stiffness, as well as on the behaviour of other couplings, and the noise frequency. It may also indicate that such a placement of this coupling element is not beneficial for the overall performance.

In Table 3, efficiency factors are calculated for MFC1, depending on the number of solenoids and the value of duty cycle of a PWM signal.

In each scenario, the most efficient value of duty cycle is 99%. The efficiency factor value is sligthly higher in scenarios of four or five couplings activated, than in scenario of one activated coupling, but these three values are similar. However, in each considered setup, eficiency factor calculated for the reference scenario also exceeds 20%. It may lead to an observation that placement of coupling element no. 1 may not be optimal, as changing its stiffness does not improve the structure performance significantly. If the duty cycle value is between 25% and 75%, efficiency factors are worse in comparison to the reference scenario and to the scenario for the duty cycle equal to 99%. However, while stiffness of the couplings is low, it may still lead to the best performance at some frequencies.

### 4.2. Coupling Point No. 2

In Figure 7, different PSD estimates of the input signals from MFC2 are presented, in the scenarios of one coupling activated (Figure 7a), four couplings activated (Figure 7b), and five couplings activated (Figure 7c), respectively. In each scenario, four different values of duty cycle of a PWM signal are considered: 25%, 50%, 75%, and 99%. As the reference scenario, PSD estimate of MFC2 input signal in case of all couplings being inactive is included in every figure.

Compared to Figure 6, more and wider frequency ranges of vibration reduction are observed for the most efficient scenarios. In Figure 7b,c, similar frequency ranges around 100–225 Hz of worse performance are observed, where the duty cycle equal to 99% causes an increase of PSD estimate level. In the mentioned frequency ranges, it is observed that the lower duty cycle values provide better results. It is not noticeable in the scenario of one activated coupling (Figure 7a).

In Table 4, efficiency factors are calculated for MFC2, depending on the number of solenoids and the value of duty cycle of a PWM signal.

The values of the best efficiency factors are higher than in the case of coupling element no. 1 (Table 3), even more than 10% for two of three scenarios (when four or five solenoids are activated). It may suggest, that the actual placement of coupling element no. 2 is closer to optimal, than the placement of coupling element no. 1. The difference of about 10% between the best efficiency factors in scenario of one activated coupling and in scenario of four or five activated couplings leads to a conclusion, that presence of other couplings between the panels is beneficial for coupling element no. 2.

### 4.3. Coupling Point No. 3

In Figure 8, different PSD estimates of the input signals from MFC3 are presented, in the scenarios of one coupling activated (Figure 8a), four couplings activated (Figure 8b), and five couplings activated (Figure 8c), respectively. In each scenario, four different values of duty cycle of a PWM signal are considered: 25%, 50%, 75%, and 99%. As the reference scenario, PSD estimate of MFC3 input signal in case of all couplings being inactive is included in every figure.

A common frequency range (about 250–300 Hz), where the performance for duty cycle equal to 99% is worse than for the other scenarios, is observed in each figure. If only the central coupling element is activated, overall performance for the scenario with the duty cycle equal to 99% is better than in Figure 8b,c, and the frequency ranges of vibration reduction are wider. Hence, in case of this coupling element, an increase of other couplings’ stiffness lowers the double-panel structure performance in this area.

In Table 5, efficiency factors are calculated for MFC3, depending on the number of solenoids and the value of duty cycle of a PWM signal. For the central coupling element, the most efficient scenario is to activate it with the duty cycle equal to 99%, as it provides maximal vibration reduction for over 43% of the frequencies in the considered range. If the central coupling is the only one not activated, a value of the best efficiency factor decreases in comparison to other scenarios.

### 4.4. Coupling Point No. 4

In Figure 9, different PSD estimates of the input signals from MFC4 are presented, in the scenarios of one coupling activated (Figure 9a), four couplings activated (Figure 9b), and five couplings activated (Figure 9c), respectively. In each scenario, four different values of duty cycle of a PWM signal are considered: 25%, 50%, 75%, and 99%. As the reference scenario, PSD estimate of MFC4 input signal in case of all couplings being inactive is included in every figure.

In Figure 9a, many narrow frequency ranges are observed, where scenario with the duty cycle equal to 99% is the most efficient one. It is a setup with only central coupling activated. Hence, coupling element no. 4 has only a role of mass loading in this scenario. However, due to change of structure stiffness in a different area, vibration reduction is observed at many frequencies. Activation of coupling no. 4 lowers the overall performance of the structure in the scenario of duty cycle equal to 99%. In contrary to the previously analyzed couplings, the common range of worse performance is not observed in three presented Figures.

In Table 6, efficiency factors are calculated for MFC4, depending on the number of solenoids and the value of duty cycle of a PWM signal. The obtained values confirm the observations based on Figure 9. If only one coupling is activated, and the duty cycle is set to 99%, the minimal PSD estimate is observed for almost 45% of the considered frequencies. The scenarios of four or five activated couplings give worse results, but their best efficiency factors are still over 30%. However, an increase of stiffness around the area of coupling no. 4 may be undesired due to decay of the double-panel structure performance. Hence, either change of coupling placement or introduction of a control system may be necessary, as well as a combination of these two solutions.

### 4.5. Coupling Point No. 5

In Figure 10, different PSD estimates of the input signals from MFC5 are presented, in the scenarios of one coupling activated (Figure 10a), four couplings activated (Figure 10b), and five couplings activated (Figure 10c), respectively. In each scenario, four different values of duty cycle of a PWM signal are considered: 25%, 50%, 75%, and 99%. As the reference scenario, PSD estimate of MFC5 input signal in case of all couplings being inactive is included in every figure.

In comparison to the results obtained for the coupling points no. 2, 3, and 4, performance of the setup with duty cycle value set to 99% is noticeably worse in each figure. The scenario of five activated couplings is also the only one in the whole analysis, where the best performance is obtained for duty cycle value set to 75%. This leads to a conclusion, that the lower stiffness of the couplings may lead to better results in some of the scenarios. However, other factors have to be taken into account, such as sensors and dampers placements, total number of couplings, etc. The widest frequency ranges for a best scenario are observed in the scenario of four activated couplings (Figure 10b).

In Table 7, efficiency factors are calculated for MFC5, depending on the number of solenoids and value of duty cycle of a PWM signal. The obtained values confirm the observations based on Figure 10. None of the values of the best efficiency factors exceed 30%, and their values are similar to each other. In the scenario of four couplings activated, efficiency increases with the increasing value of the duty cycle.

### 4.6. Comparison for All Couplings

In 14 of 15 scenarios, the most efficient duty cycle value is 99%. Table 8 presents separate comparison of efficiency factors achieved for each coupling if the duty cycle was set to 99%, to provide a different view on presented results. The highest value in each row is in boldface. An average value is calculated from each row and column.

Two out of three of the bold values are related to coupling 2, and the last one is related to coupling 4. These two elements are located in the corner areas of the panels, diagonally relative to each other (Figure 3). For these two elements, all of the values in Table 8 exceed 30%. In scenario of coupling 3, its efficiency is most noticeable if only this coupling is activated.

Average values calculated for each row of Table 8 indicate that the most efficient setup is when one coupling is activated. The worst result is obtained for five activated couplings, which may suggest negative influence of suboptimal placements of part of the couplings on the frequency band where vibration reduction is observed.

Average values calculated for each column of Table 8 show that, in general, the best results are observed around couplings numbered 2, 3 and 4. Couplings 1 and 5 give worse performance when activated, hence it may be necessary to change their placements for a better overall performance of the structure. It may also influence the structure performance, as each coupling influences the other ones. However, the effect of coupling on the mass-air-mass resonance is crucial.

In future research, an optimization algorithm will be applied to select the most efficient couplings’ configuration.

## 5. Conclusions

This paper presents a modification of the double-panel structure by coupling the panels with solenoids where vibrations of the radiating plate were measured with the use of MFCs. The concept behind introduced modification was to change vibroacoustic properties of the double-panel structure, to improve vibration isolation and/or noise reduction. Such an effect was supposed to be achieved through indirect change of couplings’ stiffness, implied by change of the duty cycle of a PWM signal provided to the solenoids. The proper placements of solenoids had an important role in improving the vibration reduction performance of the structure as well.

The presented research indicates that an optimization algorithm has to be developed to find the most beneficial placements of the couplings between the double panels. Arbitrarily selected, regular distribution of couplings may provide vibration reduction in a wide frequency band, but for the best possible effects, a theoretical basis has to be developed and validated. In general, duty cycle equal to 99% provided the best effects. However, activating all couplings with maximal stiffness was not always an optimal solution, as the highest efficiency factor of all was related to a scenario of one coupling activated. It could also mean that the placement of MFC3 was close to the optimal one and the mass-air-mass resonance was mostly influenced in such scenario. The research indicates that it is necessary to provide a control algorithm which will change duty cycle of the solenoids in real time, depending on the measurements acquired by the error sensors, which may be MFCs for vibration reduction or microphones for noise reduction.

The examined experimental setup provided a possibility to reduce vibration in a wide frequency band, despite being in a preliminary stage. The results are promising, and further research may provide even better vibration reduction performance of the structure. It has been confirmed that the use of double-panel structures in noise and vibration reduction is reasonable. One of its advantages over the single panels is, as proven in this paper, the possibility of adding modifications in the air cavity between the double panels.

The presented design of the double-panel structure may be used in the casings, which enclose noise- and vibration-generating machines, to lower the overall noise transmitted through the casing as well as vibration of the casing itself. Since the structure can generally be lightweight, it can be used for various kinds of industrial devices or even household appliances.

## Figures and Tables

**Table 1:** Properties of the solenoid.

Property	Value	Unit
mass	35	g
max. voltage	12	V
max. current	1.5	A
stroke	10	mm
max. force	5	N

**Table 2:** The measurement series.

Scenario	Number of Activated Couplings	Duty Cycle [%]
Reference scenario	0	0
Second scenario	1 (C1)	25, 50, 75, 99
Third scenario	4 (C1, C2, C4, C5)	25, 50, 75, 99
Fourth scenario	5 (C1, C2, C3, C4, C5)	25, 50, 75, 99

**Table 3:** Efficiency factors calculated for MFC1, depending on the number of solenoids and the value of duty cycle of a PWM signal.

Number of Activated Solenoids, Duty Cycle [%]	Efficiency Factor [%]
0C	22.2
1C, 25%	19.4
1C, 50%	16.0
1C, 75%	15.0
1C, 99%	**27.4**
sum	100
0C	22.8
4C, 25%	11.4
4C, 50%	15.4
4C, 75%	20.6
4C, 99%	**29.8**
sum	100
0C	25.8
5C, 25%	19.4
5C, 50%	9.4
5C, 75%	15.6
5C, 99%	**29.8**
sum	100

**Table 4:** Efficiency factors calculated for MFC2, depending on the number of solenoids and the value of duty cycle of a PWM signal.

Number of Activated Solenoids, Duty Cycle [%]	Efficiency Factor [%]
0C	17.4
1C, 25%	14.2
1C, 50%	18.4
1C, 75%	16.6
1C, 99%	**33.4**
sum	100
0C	11.0
4C, 25%	14.4
4C, 50%	16.2
4C, 75%	16.0
4C, 99%	**42.4**
sum	100
0C	13.0
5C, 25%	13.0
5C, 50%	14.2
5C, 75%	19.2
5C, 99%	**40.6**
sum	100

**Table 5:** Efficiency factors calculated for MFC3, depending on the number of solenoids and the value of duty cycle of a PWM signal.

Number of Activated Solenoids, Duty Cycle [%]	Efficiency Factor [%]
0C	19.2
1C, 25%	13.6
1C, 50%	12.6
1C, 75%	11.4
1C, 99%	**43.2**
sum	100
0C	17.2
4C, 25%	15.4
4C, 50%	23.0
4C, 75%	17.8
4C, 99%	**26.6**
sum	100
0C	12.4
5C, 25%	12.0
5C, 50%	22.6
5C, 75%	21.0
5C, 99%	**32.0**
sum	100

**Table 6:** Efficiency factors calculated for MFC4, depending on the number of solenoids and the value of duty cycle of a PWM signal.

Number of Activated Solenoids, Duty Cycle [%]	Efficiency Factor [%]
0C	11.6
1C, 25%	21.6
1C, 50%	14.4
1C, 75%	7.8
1C, 99%	**44.6**
sum	100
0C	14.2
4C, 25%	12.6
4C, 50%	17.6
4C, 75%	21.8
4C, 99%	**33.8**
sum	100
0C	21.2
5C, 25%	18.8
5C, 50%	13.4
5C, 75%	15.6
5C, 99%	**31.0**
sum	100

**Table 7:** Efficiency factors calculated for MFC5, depending on the number of solenoids and the value of duty cycle of a PWM signal.

Number of Activated Solenoids, Duty Cycle [%]	Efficiency Factor [%]
0C	16.6
1C, 25%	19.0
1C, 50%	18.6
1C, 75%	17.6
1C, 99%	**28.2**
sum	100
0C	9.8
4C, 25%	15.0
4C, 50%	20.0
4C, 75%	26.6
4C, 99%	**28.6**
sum	100
0C	20.0
5C, 25%	14.0
5C, 50%	18.2
5C, 75%	**26.6**
5C, 99%	21.2
sum	100

**Table 8:** Efficiency factors (EFs) for all couplings, depending on the number of solenoids, for duty cycle equal to 99%. Average values (avg) of columns and rows are included.

Scenario	C1 EF [%]	C2 EF [%]	C3 EF [%]	C4 EF [%]	C5 EF [%]	Avg [%]
1C, 99%	27.4	33.4	43.2	**44.6**	28.2	**35.4**
4C, 99%	29.8	**42.4**	26.6	33.8	28.6	32.2
5C, 99%	29.8	**40.6**	32.0	31.0	21.2	30.9
**avg [%]**	29.0	**38.8**	33.9	36.5	26.0	

## Abbreviations

The following abbreviations are used in this manuscript:

MFC	Macro-Fiber Composites
PSD	Power Spectral Density
PWM	Pulse Width Modulation
SSD	Synchronized Switch Damping
